# Comparative Physiological Analysis Reveals the Role of NR-Derived Nitric Oxide in the Cold Tolerance of Forage Legumes

**DOI:** 10.3390/ijms20061368

**Published:** 2019-03-19

**Authors:** Peipei Zhang, Shuangshuang Li, Pengcheng Zhao, Zhenfei Guo, Shaoyun Lu

**Affiliations:** 1 State Key Laboratory for Conservation and Utilization of Subtropical Agro-bioresources, Guangdong Engineering Research Center for Grassland Science, College of Life Sciences, South China Agricultural University, Guangzhou 510642, China; zpp198904@163.com (P.Z.); shshl1988@126.com (S.L.); 2 College of Grassland Science, Nanjing Agricultural University, Nanjing 210095, China; zpc365@163.com

**Keywords:** antioxidants, cold acclimation, freezing tolerance, *Medicago falcata*, *Medicago truncatula*, nitrate reductase, nitric oxide

## Abstract

The role of nitric oxide (NO) signaling in the cold acclimation of forage legumes was investigated in this study. *Medicago sativa* subsp. *falcata* (L.) Arcang. (hereafter *M. falcata*) is a forage legume with a higher cold tolerance than *Medicago truncatula*, a model legume. Cold acclimation treatment resulted in increased cold tolerance in both *M. falcata* and *M. truncatula*, which was suppressed by pretreatment with tungstate, an inhibitor of nitrate reductase (NR), and 2-phenyl-4,4,5,5-tetramethylimidazoline-1-oxyl 3-oxide (PTIO), a scavenger of NO. Likely, *NITRATE REDUCTASE 1* (*NIA1*), but not *NIA2* transcript, NR activity, and NO production were increased after cold treatment. Treatments with exogenous NO donors resulted in increased cold tolerance in both species. Superoxide dismutase (SOD), catalase (CAT), and ascorbate-peroxidase (APX) activities and *Cu,Zn-SOD2*, *Cu,Zn-SOD3*, *cytosolic APX1* (*cAPX1*), *cAPX3* and *chloroplastic APX1* (*cpAPX1*) transcript levels were induced in both species after cold treatment, which was suppressed by tungstate and 2-phenyl-4,4,5,5-tetramethylimidazoline-1-oxyl 3-oxide (PTIO). Treatment with exogenous NO resulted in enhanced activities of SOD, CAT, and APX. Moreover, higher levels of *NIA1* transcript, NR activity, NO production, and antioxidant enzyme activities and transcripts were observed in *M. falcata* as compared with *M. truncatula* after cold treatment. The results suggest that NR-derived NO production and upregulated antioxidant defense are involved in cold acclimation in both species, while the higher levels of NO production and its derived antioxidant enzymes are associated with the higher cold tolerance in *M. falcata* as compared with *M. truncatula*.

## 1. Introduction

Low temperature is one of the major abiotic stresses limiting plant growth and development. Temperate plants have evolved a mechanism known as cold acclimation, by which they respond to low, but non-freezing temperatures to increase their freezing tolerance [1,2]. Cold acclimation involves altered expression of thousands of genes that leads to metabolism rearrangement and physiological adaptation. A few hundred of the approximately 4000 cold-regulated (*COR*) genes are regulated by the CRT binding factors (CBFs) pathway, while the others are CBF-independent in *Arabidopsis* [2]. Some of the *COR* genes encode key enzymes for osmolyte biosynthesis and the antioxidant defense system that lead to an accumulation of cryoprotective proteins and soluble sugars for the stabilization of the cellular osmotic potential under low temperature [3,4] and activation of antioxidant defense for scavenging of reactive oxygen species (ROS) [5]. ROS is produced when plants are exposed to low temperature as a result of inhibition of the enzymes in the Calvin-Benson cycle of photosynthesis under low temperatures, which reduces the utilization of absorbed light energy for CO_2_ assimilation and results in an increased photosynthetic electron flux to O_2_ [6]. The antioxidant defense system includes superoxide dismutase (SOD), catalase (CAT), ascorbate peroxidase (APX), glutathione reductase (GR), and non-enzyme antioxidants, such as ascorbate (AsA) and glutathione (GSH). Numerous investigations reveal that the antioxidant defense system protects plants against oxidative damages induced by cold stress [5].

Substantial evidence reveals that NO participates in cold acclimation and freezing tolerance [7,8,9,10,11]. Cold induces NO production in plants, which is considered a general response in plants [12]. NO accumulates rapidly in *Arabidopsis* and *Brassica juncea* when plants are exposed to low temperatures [8,9], while treatment with exogenous NO donor increases the cold tolerance of maize by affecting antioxidant enzymes [13]. Nitric reductase (NR) is considered to be the most important enzymatic source of NO from nitrite reduction [14] and plays a key role in controlling NO levels in plants [15]. NR-dependent NO levels are positively correlated with cold acclimation and freezing tolerance in *Arabidopsis* [8,12,16]. Treatment with exogenous NO donors increases antioxidant enzyme activity in tobacco [17], and NO depletion diminishes the cold-induced expression of *CBF1*/*3* and CBF regulons, such as *COR15a*, *LTI30*, and *LTI78* in *Arabidopsis* [8]. Cold induces S-nitrosylation of some proteins. NO-mediated S-nitrosylation of iron-containing SOD is also important for chilling tolerance in *B. juncea* [11]. Recently, NR was found to supply NADH electrons to a molybdo enzyme named NOFNiR (nitric oxide-forming nitrite reductase) that catalyzes the NO production from nitrite in *Chlamydomonas* in the presence of nitrate [18]. NR is encoded by two genes, *NIA1* and *NIA2*, in *Arabidopsis* [19,20] and *M. truncatula* [21]. *NIA1* is more related to NO production than *NIA2* in *Arabidopsis* [19,20]. NR-derived NO production with induced *NIA1* expression is involved in cold acclimation in *Arabidopsis* [12]; however, it is unknown whether NR-derived NO is involved in the regulation of antioxidant enzymes during cold acclimation in legume crops.

Alfalfa (*Medicago sativa* L.) is the most important forage legume, with high biomass productivity and an excellent nutritional profile. *M. falcata*, being closely related to alfalfa and *M. truncatula*, has been used to cross with alfalfa to lead to heterosis for biomass yield due to its great tolerance to cold and drought [22]. *M. truncatula* is a legume model with low cold tolerance for the investigation of other leguminous plants [23]. It is interesting to analyze the differential response to cold between *M. falcata* and *M. truncatula* to understand the cold tolerance mechanisms in *M. falcata*. Compared to *M. truncatula*, more sucrose and proline are accumulated in *M. falcata* with higher activities of sucrose phosphate synthase and sucrose synthase during cold acclimation. Transcripts of *CRT binding factor* (*CBF*) and *cold acclimation specific* (*CAS*), a downstream target of CBF, are induced in both species during cold treatment, and higher levels of *CBF3*, *CAS17*, and *CAS18* are maintained in *M. falcata* than in *M. truncatula* [24]. A series of genes responsive to cold, such as myo-inositol phosphate synthase (*MfMIPS1*), hybrid proline-rich protein (*HyPRP1*), galactinol synthase (*MfGolS1*), inositol transporter-like (*INT-like*), S-adenosylmethionine synthetase (*MfSAMS1*), and temperature induced lipocalin (*MfTIL1*), have been identified in *M. falcata* in our laboratory [25,26,27,28,29,30]. NO is involved in cold-induced expression of *MfMIPS1*, *MfHyPRP1*, and *MfSAMS1* [25,26,29]. However, it is unknown whether NO signaling is associated with the differential cold tolerance between *M. falcata* and *M. truncatula*, while it is important for understanding the role of NO in cold acclimation as well as improvements of cold tolerance in legume crops using the genes associated with NO signaling.

The aims of this study were to investigate the relationship between cold tolerance and NO levels through a comparison of the physiological responses to cold in *M. falcata* with those in *M. truncatula*, and to elucidate the key role of NO in the regulation of cold tolerance in forage legumes. We found that NR-derived NO production is involved in the cold acclimation of *M. falcata*, at least through regulating antioxidant enzymes.

## 2. Results

### 2.1. Differential Cold Tolerance in M. falcata and M. truncatula

**Figure 1:**
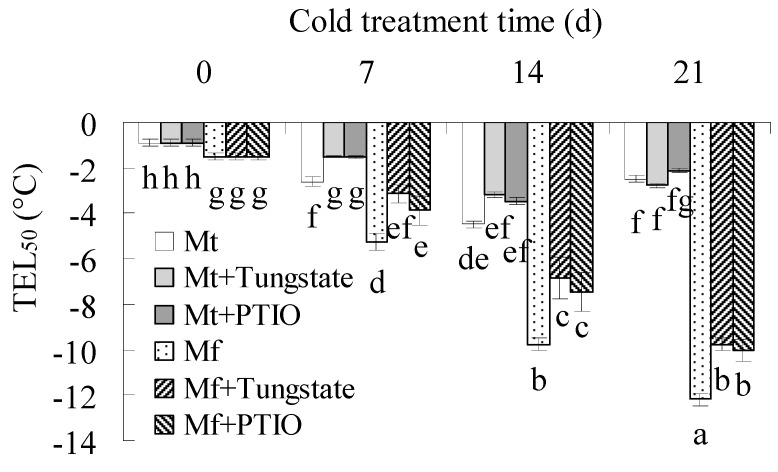
Cold tolerance in *M. falcata* (Mf) in comparison to *M. truncatula* (Mt) as affected by tungstate and 2-phenyl-4,4,5,5-tetramethylimidazoline-1-oxyl 3-oxide (PTIO) during 21 d of cold treatment. Ten-week-old *M. falcata* and *M. truncatula* cv. A17 plants were irrigated with 15 mL of 1 mM tungstate or 100 μM PTIO solution or H_2_O as a control, followed by exposure to 5 °C in a growth chamber. Ion leakage of leaves was measured to calculate the temperature that resulted in 50% lethality (TEL_50_). Means of three replicates and standard errors are presented; the same letter below the column indicates no significant difference at *p* < 0.05.

**Figure 2:**
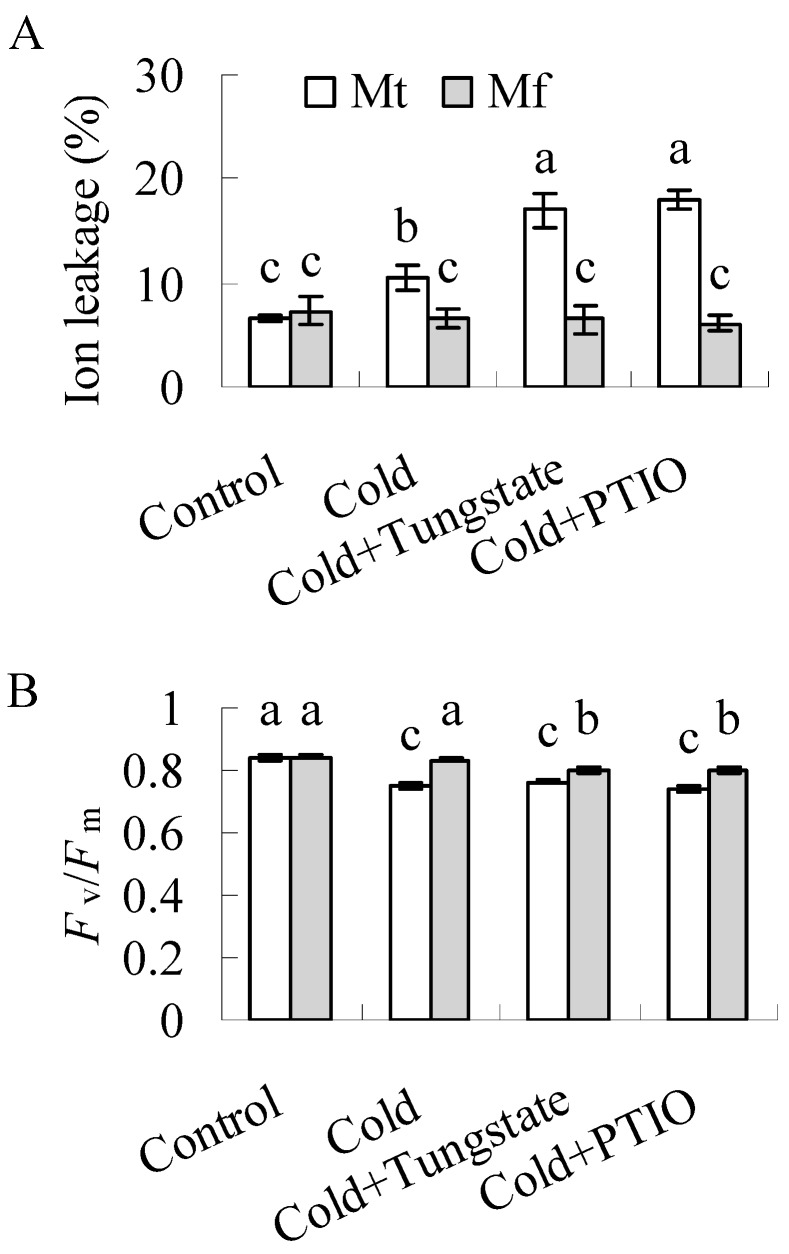
Ion leakage (**A**) and maximum photochemistry efficiency of photosystem II (*F_v_*/*F*_m_, **B**) in *M. falcat*a (Mf) in comparison to *M. truncatula* (Mt) as affected by tungstate and PTIO after cold treatment. Ten-week-old *M. falcata* and *M. truncatula* cv. A17 plants were irrigated with 15 mL of 1 mM tungstate or 100 μM 2-phenyl-4,4,5,5,-tetramethylimidazoline-1-oxyl 3-oxide (PTIO) solution or H_2_O as a control, followed by exposure to 5 °C in a growth chamber for 21 d. Means of three replicates and standard errors are presented; the same letter above the column indicates no significant difference within each day at *p* < 0.05.

A temperature that results in 50% electrolyte leakage (TEL_50_) was determined for the evaluation of cold tolerance [23]. Lower TEL_50_ was observed in non-acclimated plants of *M. falcata* than in *M. truncatula*. TEL_50_ was decreased in both *M. falcata* and *M. truncatula* during cold treatment at 5 °C, except for that in *M. truncatula* at 21 d, and lower TEL_50_ was observed in *M. falcata* than in *M. truncatula* throughout the cold treatment (Figure 1), indicating that *M. falcata* had higher cold tolerance than *M. truncatula*. The increased TEL_50_ at 21 d in *M. truncatula* indicates a decrease in the cold tolerance. To understand whether this case was associated with chilling injury as a result of the long time exposure to low temperatures, the ion leakage and *F*_v_/*F*_m_ were measured. Compared to control plants, ion leakage was increased and *F*_v_/*F*_m_ was decreased in *M. truncatula* after 21 d of cold treatment, while they were not altered in *M. falcata* (Figure 2A,B). The results indicated that *M. truncatula* plants were damaged by long time exposure to low temperatures as compared with *M. falcata*., which led to a decreased freezing tolerance in *M. truncatula*.

### 2.2. NO is Involved in Cold Acclimation of M. falcata and M. truncatula

**Figure 3:**
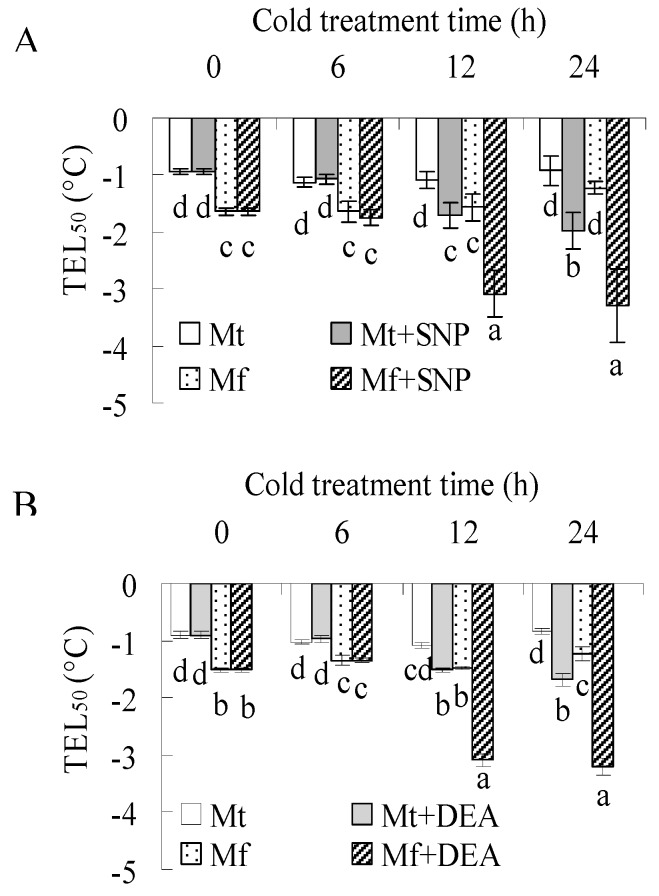
Cold tolerance in *M. falcata* (Mf) in comparison to *M. truncatula* (Mt) as affected by treatment with exogenous nitric oxide generators. Detached leaves of *M. falcata* and *M. truncatula* cv. A17 plants were treated with 200 μM sodium nitroprusside (SNP, **A**), 100 μM diethylamine diethylammonium (Z)-1-(N,N-diethylamino) diazen-1-ium-1,2-diolate (DEA, **B**), or H_2_O as a control. Ion leakage of leaves was measured to calculate the temperature that resulted in 50% lethality (TEL_50_). Means of three replicates and standard errors are presented; the same letter above the column indicates no significant difference within each day at *p* < 0.05.

Pretreatment with PTIO or tungstate suppressed the decrease of TEL_50_ in both *M. falcata* and *M. truncatula* during cold treatment (Figure 1), implying that NR-derived NO is associated with cold acclimation in both species. The data also showed that TEL_50_ was continuously reduced in PTIO- or tungstate-treated plants during cold treatment, indicating that PTIO or tungstate could not fully block cold acclimation. To confirm the role of NO, the effect of the exogenous generator of NO on cold tolerance was examined. Leaflets were incubated with SNP or diethylammonium (Z)-1-(N,N-diethylamino) diazen-1-ium-1,2-diolate (DEA), donors of NO production, followed by measurement of TEL_50_. The result showed that TEL_50_ was significantly decreased in both species by 12 or 24 h of treatment with SNP or DEA (Figure 3A,B), indicating that the NO level is associated with cold tolerance. The above results suggested that NR-derived NO was involved in the cold acclimation of *M. falcata* and *M. truncatula*, but other mechanisms except for NO are also essential for cold acclimation.

### 2.3. NO Production was Induced during Cold Treatment

**Figure 4:**
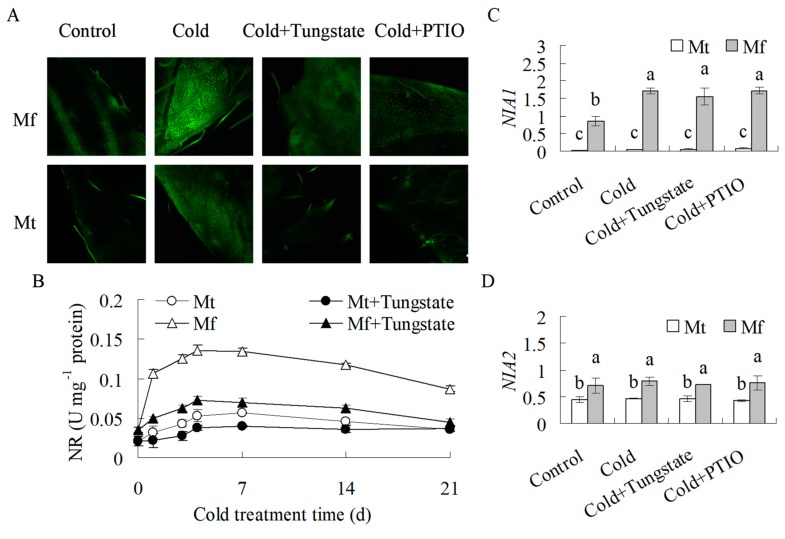
Analysis of nitric oxide (NO) production (a), nitrate reductase (NR) activity, and *NIA1* and *NIA2* transcripts in response to cold treatment in *M. falcata* (Mf) in comparison to *M. truncatula* (Mt). Ten-week-old *M. falcata* and *M. truncatula* cv. A17 plants were irrigated with 15 mL of 1 mM tungstate or 100 μM 2-phenyl-4, 4, 5, 5-tetramethylimidazoline-1-oxyl 3-oxide (PTIO) solution or H_2_O as a control, followed by exposure to 5 °C in a growth chamber. After 24 h of cold treatment, NO production was detected using the NO specific fluorescent probe, DAF-FM-DA (**A**), and *NIA1* and *NIA2* transcripts were detected using quantitative RT-PCR. *Actin* was used as a reference gene to calculate the relative expression (**C**,**D**). Scan bar is 500 μm. NR activity was detected as indicated in the figure (**B**). Means of three replicates and standard errors are presented; the same letter above the column indicates no significant difference within each day at *p* < 0.05.

A weak DAF fluorescence was observed in the leaves of both species before cold treatment, with higher fluorescence in *M. falcata* than in *M. truncatula.* The DAF fluorescence was enhanced after 24 h of cold treatment in both species, which was suppressed by pretreatment with tungstate and PTIO (Figure 4A,B), indicating that the cold-induced NO production was associated with NR. In addition, higher fluorescence was observed in *M. falcata* than in *M. truncatula* (Figure 4A,B).

Nitrate reductase activity was increased in both species during cold treatment, which was inhibited by pretreatment with tungstate. In addition, higher NR activity was maintained in *M. falcata* than in *M. truncatula* (Figure 4C), which was consistent with the NO data (Figure 4B). *NIA1* transcript levels were induced by 99% and 44%, respectively, in *M. falcata* and *M. truncatula* after 24 h of cold treatment, which were not altered by pretreatment with tungstate or PTIO (Figure 4D). The *NIA2* transcript level was not responsive to cold in both species (Figure 4E). The results indicated that the increased NR activity in *M. falcata* during cold treatment might be associated with the induced expression of *NIA1*.

### 2.4. Antioxidant Enzyme Activities were Induced by Cold and NO

**Figure 5:**
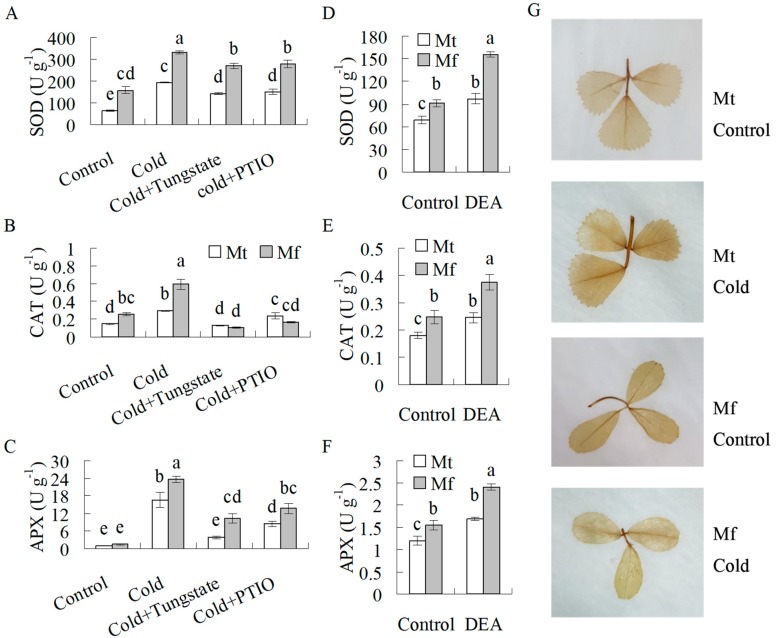
Superoxide dismutase (SOD, **A**,**D**), catalase (CAT, **B**,**E**), and ascorbate-peroxidase (APX, **C**,**F**) activities as well as H_2_O_2_ accumulation (**G**) in response to cold treatment or DEA treatment in *M. falcat*a (Mf) in comparison to *M. truncatula* (Mt). Ten-week-old *M. falcata* and *M. truncatula* cv. A17 plants were irrigated with 15 mL of 1 mM tungstate or 100 μM 2-phenyl-4,4,5,5-tetramethylimidazoline-1-oxyl 3-oxide (PTIO) solution or H_2_O as a control, followed by exposure to 5 °C for 14 d in a growth chamber (**A**–**C**). The detached leaves were treated with DEA solution for 12 h (**D**–**F**). Leaflet was detached from the control or cold treated plants (14 d) for DAB staining (**G**). Means of three replicates and standard errors are presented; the same letter above the column indicates no significant difference within each day at *p* < 0.05.

Nitric oxide has been previously documented to induce antioxidant enzyme activity and gene transcript in tobacco [31], while the antioxidant defense system plays an important role in abiotic stress tolerance. To understand whether NR-derived NO regulates antioxidant defense in *M. falcata* as compared to in *M. truncatula*, antioxidant enzyme activities were determined after 14 d of cold treatment when both species showed increased cold tolerance in response to cold treatment. Higher activities of SOD and CAT were observed in *M. falcata* than in *M. truncatula* under the control condition (Figure 5A,B). SOD, CAT, and APX activities were increased in both species after 14 d of cold treatment, and higher levels were observed in *M. falcata* than in *M. truncatula*, while pretreatment with tungstate or PTIO suppressed the increase in enzyme activities (Figure 5A–C). For further confirmation of the involvement of NO in the induction of the enzyme activities, the effects of exogenous NO on antioxidant enzymes were examined. SOD, CAT, and APX activities were induced after 12 h of treatment with DEA in both species, with higher activities in *M. falcata* than in *M. truncatula* (Figure 5D–F). The above results indicated that the induced antioxidant enzyme activities during cold treatment were associated with NR-derived NO production. H_2_O_2_ was measured after 14 d of cold treatment. Compared to the control, H_2_O_2_ accumulation was observed in *M. truncatula*, but not in *M. falcata* after 14 d of cold treatment (Figure 5G).

**Figure 6:**
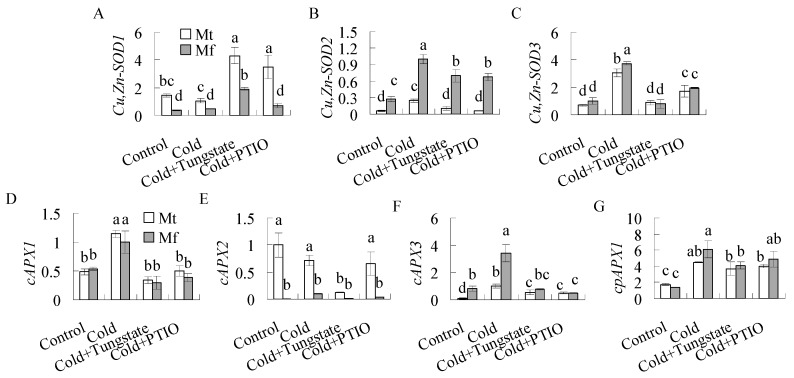
Antioxidant enzyme transcripts in response to cold treatment in *M. falcata* (Mf) in comparison to *M. truncatula* (Mt). Ten-week-old *M. falcata* and *M. truncatula* cv. A17 plants were irrigated with 15 mL of 1 mM tungstate or 100 μM 2-phenyl-4,4,5,5,-tetramethylimidazoline-1-oxyl 3-oxide (PTIO) solution or H_2_O as a control, followed by exposure to 5 °C for 24 h in a growth chamber. *Cu,Zn-SOD1* (**A**), *Cu,Zn-SOD21* (**B**), *Cu,Zn-SOD3* (**C**), *cAPX1* (*cytosolic APX*, **D**), *cAPX2* (**E**), *cAPX3* (**F**), and *cpAPX1* (*chloroplast APX*, **G**) transcripts were detected using qRT-PCR, and *actin* was amplified as a reference gene to calculate the relative expression. Means of three replicates and standard errors are presented; the same letter above the column indicates no significant difference at *p <* 0.05.

The transcript levels of the genes encoding SOD and APX were further examined. *Cu,Zn-SOD1* and *cAPX2* transcripts were not induced by cold in both species (Figure 6A,E). Transcript levels of *Cu,Zn-SOD2*, *Cu,Zn-SOD3*, *cAPX3*, and *cpAPX1* were induced after 24 h of cold treatment, which was inhibited by pretreatment with tungstate or PTIO in both species (Figure 6B,C,F,G). Although *cAPX1* transcript was induced in both species by cold treatment, but the induction was not altered by pretreatment with tungstate and PTIO (Figure 6D). The results suggest that the cold induced antioxidant enzyme activities were associated with the expression of *Cu,Zn-SOD2*, *Cu,Zn-SOD3*, *cAPX3*, and *cpAPX1*.

## 3. Discussion

*M. falcata* is important because of its great cold tolerance and is used for crossing with alfalfa in alfalfa breeding [22], while *M. truncatula* is an annual legume without a cold acclimation mechanism [23]. TEL_50_ is commonly used to evaluate the cold tolerance of alfalfa [23]. Lower TEL_50_ was observed in both non-acclimated and acclimated plants of *M. falcata* than in *M. truncatula*. Compared to the continuous decrease of TEL_50_ in *M. falcata* within 21 d of cold treatment, *M. truncatula* showed a chilling injury, with increased ion leakage and decreased *F*_v_/*F*_m_ at 21 d of cold treatment, which resulted in an increase in TEL_50_. The results indicated that *M. falcata* had higher cold tolerance than *M. truncatula*, which is consistent with a previous report [23]. The differences in the *CAS* gene copy numbers and CRT/DRE copy numbers in the *CAS* gene upstream regions are proposed to determine the differential cold tolerance between *M. falcata* and *M. truncatula* [23]. Given that NO is involved in cold-induced expression of *CBF1*/*3* and CBF regulons, such as *COR15a*, *LTI30*, and *LTI78*, in *Arabidopsis* [8] and NO production is increased in diverse plant species when plants or plant organs are exposed to low temperature [7,8,9,10,12], the importance of NO in cold tolerance is documented in the present study.

Nitrate reductase is the major enzyme catalyzing production of NO from nitrite reduction [14]. The cold-induced NO production is impaired in NR-deficient mutants or by treatments with NR inhibitors in *Arabidopsis* and *Brassica napus* [8,12]. Similar results were observed in this study. NO production, NR activity, and *NIA1* transcript were increased after low temperature treatment in both *M. falcata* and *M. truncatula*, while the increased NO production was impaired by pretreatment with an inhibitor of NR in both species. Cold acclimation led to increased cold tolerance in *M. falcata* and *M. truncatula*, which was blocked by pretreatment with an inhibitor of NR or scavenger of NO, while exogenous NO treatments increased the cold tolerance in both *M. falcata* and *M. truncatula*. The results suggest that NR derived NO is involved in the cold acclimation of *M. falcata* and *M. truncatula*. In addition, higher levels of both NR activity and NO production were observed in *M. falcata* than in *M. truncatula* under low temperature conditions. It is suggested that the difference in NR activity and NO production during cold acclimation is associated with the differential cold tolerance between *M. falcata* and *M. truncatula*. An analysis of the promoter region of *NIA1* in *M. falcata* is worthwhile to reveal the regulation of cold on NO production in the future. In addition, our results showed that cold acclimation increased cold tolerance was not completely inhibited by pretreatment with an NR inhibitor or NO scavenger, indicating that NO is not the exclusive factor for cold acclimation. NO is signaling in gene expression. The expression of a total of 1023 cDNA fragments and 1932 genes are altered in response to NO in *M. truncatula* and upland cotton, respectively [32,33], compared to the altered expression of 4000 genes during cold acclimation [2]. Thus, mechanisms other than NO signaling are also associated with the differences between *M. falcata* and *M. truncatula*.

It is unavoidable for plants to accumulate ROS under low temperature conditions, as a result of an imbalance between the production and utilization of a photo-generated reductant that leads to an increased photosynthetic electron flux to O_2_ for the production of ROS. Higher antioxidant enzyme activities, including SOD, CAT, and APX, are maintained in chilling tolerant mutants than in the wild type of centipedegrass and stylo under low temperature conditions to avoid oxidative damages [34,35]. SOD, CAT, and APX activities are induced in *M. falcata* during cold treatment, and an induced expression of the genes encoding antioxidant enzymes is associated with the enhanced cold tolerance in transgenic tobacco plants overexpressing *MfSAMS1* [29]. SOD, CAT, and APX activities and transcripts of *Cu, Zn-SOD2*, *Cu, Zn-SOD3*, *cAPX3*, and *cpAPX1* were induced in *M. falcata* and *M. truncatula* after cold treatment, which were impaired by treatments with an NR inhibitor and NO scavenger. Exogenous application of NO induced SOD, CAT, and APX activities, which was consistent with our previous observation in stylo and bermudagrass [31,36]. The results suggest that NR-derived NO is involved in cold induced antioxidant enzyme activities in *M. falcata* and *M. truncatula*. Moreover, *M. falcata* had higher activities of SOD, CAT, and APX and a lower accumulation of H_2_O_2_ than *M. truncatula* during cold treatment, suggesting that the higher activities are associated with the higher cold tolerance in *M. falcata* compared with *M. truncatula*. NO induces antioxidant enzyme expression by activating mitogen-activated protein kinase (MAPK) cascades, which is downstream of the ABA and H_2_O_2_ signaling pathway, in maize in response to water stress [37] and in bermudagrass, stylo, and tobacco plants [17,31,35,36,38]. Except for an involvement in the induction of antioxidant expression, NO also mediates cold induced expression of other genes, such as *MfMIPS1*, *MfHyPRP*, and *MfSAMS1*, and those genes confer cold tolerance in *M. falcata* [25,26,29]. NO is an important signal regulating cold acclimation in forage legumes by mediating the expression of multiple cold responsive genes. Our results did not exclude the possible role of NO in cold tolerance through S-nitrosylation. S-Nitrosoglutathione is an important in vivo S-nitrosylating agent that is formed by the reaction between NO and GSH. S-nitrosylation can activate or inhibit protein activity and affect protein translocation and function, for example, NO-mediated S-nitrosylation of iron-containing SOD is associated with chilling tolerance in *B. juncea* [11]. Cold-induced modifications of S-nitrosylation proteins have been identified in various plant species [7,11]. It remains to be determined whether S-nitrosylation is involved in the cold tolerance in *M. falcata*.

In conclusion, NO signaling plays an important role in the cold acclimation of forage legumes, such as *M. falcata* and *M. truncatula*. NR-derived NO production is involved in the cold acclimation of *M. falcata* and *M. truncatula*, by up-regulating antioxidant enzymes. Moreover, the higher levels of NR activity and NO production and its derived antioxidant enzyme activities are associated with the higher cold tolerance in *Medicago falcata* as compared with *M. truncatula*.

## 4. Materials and Methods

### 4.1. Plant Growth and Treatments

Plants of *Medicago falcata* cv. *Hulunbeir* and *Medicago truncatula* cv. A17 were grown in a mixture of peat and perlite (3:1, v/v) in plastic pots (15 cm diameter and 15 cm depth) under natural light in a greenhouse for 8–10 weeks as described previously [25,27]. Ten-week-old *M. falcata* and *M. truncatula* cv. A17 plants were divided into three groups and respectively irrigated with 15 mL of 1 mM tungstate, NR inhibitor [18], 100 μM 2-phenyl-4,4,5,5-tetramethylimidazoline-1-oxyl 3-oxide (PTIO) solution, NO scavenger [39], or H_2_O as a control, and then transferred to a growth chamber at 5 °C under light of 200 μmol photos m^−2^ s^−1^ for 1 d, 14 d, or 21 d for cold treatment before sampling for specific measurements. Plants were irrigated with the above solutions of 15 mL of the reagents once every three days. For treatment with exogenous NO generators, detached leaves of *M. falcata* and A17 were placed in a petri dish containing 100 μM diethylammonium (Z)-1-(N,N-diethylamino) diazen-1-ium-1,2-diolate (DEA), 200 μM sodium nitroprusside (SNP) [37], or H_2_O as a control, respectively, for 12 h under light of 200 μmol m^−2^ s^−1^. Each pot contained five plants. Each leaf sample was harvested from one pot, and three samples were used for measurements as replicates.

### 4.2. Isolation of RNA and Real-Time Quantitative PCR (qPCR) Analysis

Total RNA was isolated from leaves using a HiPure Plant RNA Mini Kit (Magen, Guangzhou, China). 1 μg of total RNA was used for synthesis of first-strand cDNA, using the PrimeScript RT reagent Kit with gDNA eraser (Takara Bio Inc., Otsu, Shiga, Japan). qPCR was conducted for the detection of gene transcripts in a Mini Option Real-Time PCR system (Bio-Rad, Hercules, CA). PCR solution (10 μL) contained 15 ng of diluted cDNA template, 200 nM each for forward and reverse primers, and 5 μL SYBR Premix Ex Taq (Takara Bio Inc.). The primers and sequences are listed in Table 1. A negative control without a cDNA template was always included. Parallel reactions to amplify *actin1* were used to normalize the amount of template, as *actin1* is reliable as z reference gene in *M. falcata* and *M. truncatula* [24]. Melting profiles were detected for validation of the primer specificity, showing a single product specific melting temperature. All PCR efficiencies were above 95%. Relative expression was calculated by 2^−ΔΔCt^, which was done automatically by the instrument.

### 4.3. Evaluation of Cold Tolerance

Cold tolerance was estimated by the temperature that resulted in 50% ion leakage (TEL_50_) [23]. After plants were exposed to low temperature at 5 °C for 0, 7, 14, and 21 d (Figure 1), or 0, 6, 12, and 24 h, leaflets were detached and placed in a tube, which was then placed on ice for 1 h of equilibrium, followed by the addition of ice chips to the leaflets in each tube. The tubes were then placed in an ethanol bath in a programmable freezer (model: Polystat cc1 and k6, Huber Unit, Offenburg, Germany), followed by equilibration for 1 h at 0 °C and the temperature was decreased at a rate of −2 °C h^−1^ to various freezing temperatures (0, −2, −4, −6, −8, −10, −12, and −14 °C) with 1 h of holding. Three tubes were used as a replicate at each temperature. After thawing overnight at 0 °C in a freezer, 6 mL of deionized water was added to each tube. Ion leakage was measured at room temperature and calculated as (*C*_1_/*C*_2_) × 100, where *C*_1_ and *C*_2_ indicate the conductivity before and after heating as previously described [25]. The freezing temperatures and corresponding ion leakages were used for calculation of TEL_50_ using a logistic sigmoid fitted model plot by software Origin 9.0 (OriginLab, Hampton, 01036). For the evaluation of the chilling tolerance, the ion leakage and the maximum photochemical efficiency of the photosystem II (*F*_v_/*F*_m_) was measured after plants were exposed to a low temperature of 5 °C for 21 d. For measurement of the ion leakage, leaflets were placed in a tube, followed by addition of 6 mL of deionized water. Ion leakage was measured at room temperature and calculated as (*C*_1_/*C*_2_) × 100, where *C*_1_ and *C*_2_ indicate the conductivity before and after heating as previously described [25]. *F*_v_/*F*_m_ was measured as described previously using a pulse-modulated fluorometer (Model FMS-2, Hansatech Instruments) according to the manufacturer’s instructions [35].

### 4.4. Detection of NO in Leaves

NO-specific fluorescent dye 4-amino-5-methylamino-2,7-difluorofluorescein diacetate (DAF-FM DA, Sigma) was used for the determination of NO production as described previously [12] with modifications. Leaflets were incubated in 10 mM Tris-HCl buffer (pH 7.0) containing 10 μM DAF-FM for 1 h in the dark at room temperature, followed by washing with Tris-HCl (pH 7.0) buffer. A confocal laser scanning microscope (LSM 510, Carl Zeiss, Jena, Germany) was used for imaging the leaflets, with excitation at 488 nm and emission at 515 nm. The images were processed and analyzed using Image J software (Wayne Rasband, NH, USA). Data are presented as the relative fluorescence intensity [40].

### 4.5. Determination of NR, SOD, CAT, and APX Activities

Nitrate reductase, SOD, and CAT were extracted from leaves (0.3 g) in 3 mL of 50 mM phosphate buffer (pH 7.8) containing 2% (*w/v*) PVP and 2 mM EDTA, while APX was extracted in 3 mL of 50 mM phosphate buffer (pH 7.0) containing 2% (*w/v*) PVP, 1 mM AsA, and 1 mM EDTA. After centrifugation at 15,000 × g for 15 min at 4 °C, the supernatants were recovered for determinations of NR, SOD, CAT, and APX activity as previously described [17]. One unit of NR was defined as the amount of enzyme required to catalyze the conversion of 1 μmol NO_3_^−^ within 1 h, while one unit of CAT or APX was defined as the amount of enzyme required to catalyze the conversion of one μmol H_2_O_2_ (extinction coefficient 0.00394 mM^−1^ cm^−1^) or ascorbic acid (extinction coefficient 2.8 mM^−1^ cm^−1^) within 1 min. One unit of SOD activity was defined as the amount of enzyme required for inhibition of the photochemical reduction of ρ-nitro blue tetrazolium chloride by 50%.

### 4.6. Statistical Analysis

The experimental data were subjected to an analysis of variances using an SPSS program (SPSS Inc., Chicago, IL). Duncan’s t-test was used to evaluate the differences among the means of treatments and plant lines at the 0.05 probability level.

## Figures and Tables

**Table 1:** Primers used for real-time quantitative PCR (qPCR).

Gene name	Accession No.	Primer name	Sequence
*MtNIA1*/*MfNIA1*	MTR3g073180	ZG2619	TGGCTCAACCTTGGATAT
		ZG2620	TGCTTACCGTGAACCATA
*MtNIA2*/*MfNIA2*	MTR5g059820	ZG2621	GAGGATTGTTGTTACTACTG
		ZG2622	CACGGAGTTTATGTTCA
*MtCu,Zn-SOD1/MfCu,Zn-SOD1*	MTR4g057240	ZG6010 ZG6011	GCTTAATGTCCTAGATGAGT AGCAGGCAAGAAGTATTG
*MtCu,Zn-SOD2/MfCu,Zn-SOD2*	MTR6g029200	ZG6012 ZG6013	ACTCCAGTCATCTGTTTAG CCATTACGCATAGAACAAC
*MtCu,Zn-SOD3/MfCu,Zn-SOD3*	MTR7g114240	ZG6014 ZG6015	TTCCATATCCATGCCTTG CGTGCTCCTTACCATTAG
*MtcAPX1*/*MfcAPX1*	MTR3g107060	ZG6016	GGAGGTCCTACTATCACA
		ZG6017	CCAGAAGCATCAAGAGTT
*MtcAPX2*/*MfcAPX2*	MTR4g061140	ZG6020	CCTGATGGAGTGTTCAAT
		ZG6021	ACCTTGCCTACTTCATATC
*MtcAPX3*/*MfcAPX3*	MTR5g022510	ZG6022	GATAAGGCTCTAGTTGATGA
		ZG6023	TGAGGCAATAACCATTCC
*MtcpAPX1*/*MfcpAPX1*	MTR3g088160	ZG6024	TGGATTCTGAACAGTGAAC
		ZG6025	CGACCTCCTCTATCTTGA
*MtActin*/*MfActin*	MTR3g095530	ZG1613	ATTCACGAGACCACCTAC
		ZG1614	GAGCCACAACCTTAATCTTC
